# 790. Evaluation of an Enhanced CPE Screening Program in an Acute Care Hospital in South Korea

**DOI:** 10.1093/ofid/ofab466.987

**Published:** 2021-12-04

**Authors:** Sun Hee Park, Yunmi Yi, Seul Ki Ji, Seung Beom Han, Soyoung Shin

**Affiliations:** 1 College of Medicine, the Catholic University of Korea, Daejeon, Taejon-jikhalsi, Republic of Korea; 2 Daejeon St Mary's Hospital, Daejeon, Taejon-jikhalsi, Republic of Korea; 3 Daejeon St. Mary's Hospital, Daejeon, Taejon-jikhalsi, Republic of Korea

## Abstract

**Background:**

Carbapenemase-producing Enterobacteriaceae (CPE) poses a great challenge in infection control in healthcare settings. A screening and contact precautions are recommended to prevent the spread of CPE among patients. However, screening strategies differ among countries and healthcare facilities.

**Methods:**

In September 2018, we launched a CPE screening program at a 660-bed hospital in South Korea, which targeted previously colonized patients, patients with history of admission < 1 month or transferred patients or ICU-admitted patients. Once patients were identified to have CPE, they were isolated in a single room. After a CPE outbreak in July-Aug 2019, the enhanced screening program was implemented, which included patients with additional risk factors (exposure to hospitals in the past 6 months, receipt of hemodialysis or invasive procedures or rehabilitation) combined with weekly screening in ICU-admitted patients. Screening methods changed from two consecutive rectal screening swabs with chromogenic agar to initial screening with Xpert-Carba-R PCR, followed by one or two consecutive tests with chromogenic agar. We compared the CPE incidence in screening and clinical cultures before and after the enhanced screening program introduction (Sep 2018-Nov 2020).

**Results:**

A total of 14,318 (2,178 vs. 12,140) were screened among 49,980 admitted patients and screening compliance increased from 18.6% to 94.5%. The number of CPE detection increased from 44 to 154 cases and the proportion of CPE-positive screening per 1000 admissions increased 0.6 to 2.2. However, the number of clinical CPE cultures decreased from 11 to 3 (Figure). Among screened patients, time-to-positivity was markedly reduced by 1.9 days (2.96 vs. 1.02 days) during the post-period. Additional 70 patients were detected: 36 due to serial screening in the ICUs and 34 due to enhanced on-admission screening. Factors significantly associated with positive screening were previous exposure to hospital (OR 3.5; 95% CI 1.7-7.1) and receipt of hemodialysis (OR 4.3; 95%CI 1.9-9.2). CPE isolates and carbapenemase genes were diverse (Figure). Trends in CPE detection in screening and clinical samples (upper), and bacterial species with detected carbapenemase genes (lower).

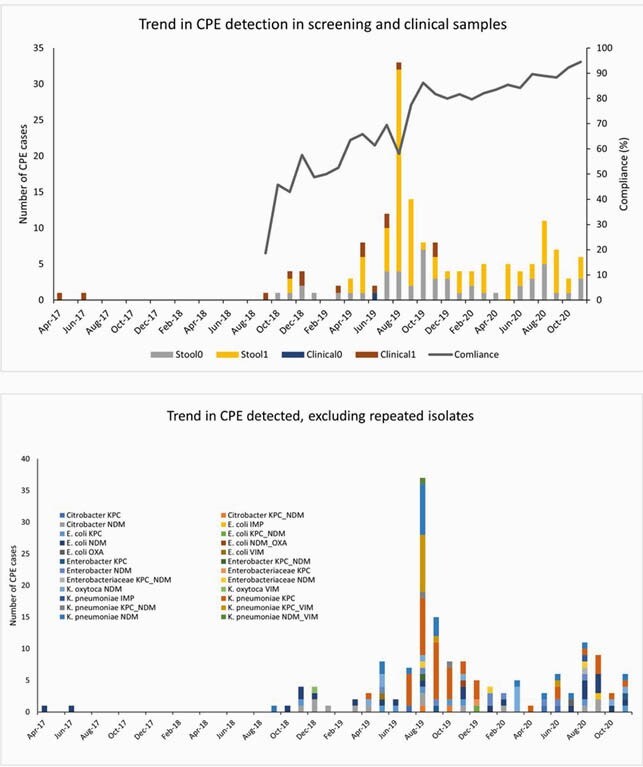

**Conclusion:**

The study results showed that the enhanced screening program enabled us to identify the previously undetected CPE colonized patients and to decrease clinical CPE cultures.

**Disclosures:**

**All Authors**: No reported disclosures

